# QT Interval Dispersion as a Predictor of Clinical Outcome in Acute Ischemic Stroke

**DOI:** 10.3389/fneur.2020.00974

**Published:** 2021-01-22

**Authors:** Hefei Tang, Jiayao Sun, Yu Wang, Xu Jie, Yan Ma, Anxin Wang, Yijun Zhang, Xingao Wang, Yongjun Wang

**Affiliations:** ^1^Department of Neurology, Beijing Tiantan Hospital, Capital Medical University, Beijing, China; ^2^China National Clinical Research Center for Neurological Diseases, Beijing, China; ^3^Center of Stroke, Beijing Institute for Brain Disorders, Beijing, China; ^4^Beijing Key Laboratory of Translational Medicine for Cerebrovascular Disease, Beijing, China; ^5^Department of Neurology, Zhangjiakou First Hospital, Hebei, China; ^6^Division of Cardiology, Department of Internal Medicine, Beijing Tiantan Hospital, Capital Medical University, Beijing, China

**Keywords:** acute ischemic stroke, functional outcome, QT dispersion, recurrent vascular event, TIA

## Abstract

**Background and Purpose:** QT dispersion (QTd) abnormalities are widely documented in stroke patients. This study aims to investigate the association between QTd and clinical outcomes in IS patients.

**Methods:** IS patients registered in the Blood Pressure and Clinical Outcome in transient ischemic attack (TIA) or IS (BOSS) registry between 2012 and 2014 within 24 h of onset were analyzed. In this prospective observational study, we identified 1,522 IS cases with adequate electrocardiographic evaluations to assess QTd after the index stroke. Patients were classified into four groups based on the quartile of QTd, with the lowest group as the reference. The primary stroke outcome was defined as a modified Rankin Scale score ≥3 at 1-year. Multiple logistic regressions were utilized to investigate the association between QTd and outcome events.

**Results:** The mean QTd across all cases was 57 ms (40–83). Functional dependency or death was documented in 214 (14.98%) cases at 1 year. After adjusting for confounders, the prevalence of death and major disability (mRS ≥ 3) showed significant differences according to the quartile of QTd, with the risk of death and major disability (mRS ≥ 3) at 1 year being significantly higher for patients in Q4 than for those in Q1 (adjusted OR = 1.626, 95% CI:1.033–2.560). However, there were no significant correlation between QTd and the event outcomes at 1 year.

**Conclusions:** QTd was associated with poor functional outcomes at 1 year. QTd is a useful surrogate marker for adverse functional prognosis, which might help to stratify risk in patients with acute IS.

## Introduction

Stroke is one of the most common causes of death and disability (1). A huge variety of factors are known to influence patient outcome, including demographic variables, clinical variables, laboratory tests, or comorbidities (2). But predicting the final neurological outcomes is very difficult after the index stroke because most studies presenting contradictory results (3, 4).

Patients with acute stroke are still at risk for adverse clinical outcomes, as the treatment primarily focuses on neurological recovery and ignores the hierarchical management of cardiovascular complications (5). Furthermore, many stroke survivors are less likely to exercise enough to develop the significant symptoms of cardiac disease due to movement disorders or complete the traditional cardiac examination for risk stratification. Alternative approaches and novel thinking are therefore required in stroke survivors.

Lesions in the central nervous system often cause autonomic dysregulation (6). The major autonomic dysfunctions caused by ischemic stroke (IS) include a loss of heart rate variability and various ECG changes, particularly QT dispersion (QTd), which is an expression of cardiac repolarization abnormalities (7). Several studies have confirmed the association between acute cerebrovascular events and QTd (8–12). In patients admitted to hospital for acute cerebrovascular diseases, QTd may reflect neurologic injury as well as the underlying heart disease. Thus, it can be used as a marker of adverse clinical prognosis after acute ischemic stroke. Unfortunately, many of these early studies either did not differentiate between hemorrhagic and ischemic stroke or were single-center studies with small sample sizes, making it difficult to draw firm conclusions. Furthermore, the existing data regarding the effect of QTd on the long-term outcomes of these patients are contradictory. Therefore, we aimed to assessed whether abnormal QTd are associated with adverse prognosis of patients with acute IS in the BOSS (blood pressure and clinical outcome in transient ischemic attack [TIA] or IS) study.

## Patients and Methods

### Study Design and Population

This study was conducted among patients from the BOSS study (a nationwide, hospital-based, longitudinal cohort) consecutively enrolled from October 2012 to February 2014 at 61 hospitals in China. The design of the BOSS study has been described in detail elsewhere (13). The BOSS registry inclusion criteria were as follows: age 18 years or older; diagnosis of an acute IS or TIA; and recruited within 7 days of symptom onset. Baseline information about the following risk factors was collected: hypertension, diabetes, dyslipidemia, current, or previous smoking, and moderate or heavy alcohol consumption (≥2 standardized alcohol drinks per day). In this study, those who had acute myocardial infarction (*n* = 10), atrial fibrillation (*n* = 73), bundle branch block (*n* = 77), atrioventricular block (*n* = 52), or a history of stroke (*n* = 618) were excluded. After exclusions, our final sample size included 1,841 participants. The protocol and data collection of the trial were approved by the ethics committee of Beijing Tiantan Hospital and all participating centers. All participants provided written informed consent.

### Measurement of QTD

For each patient, standard 12-lead-ECGs (paper speed of 25 mm/s, standardization of 10 mm/1 mV) were recorded at admission and retrieved and analyzed manually with caliper by two independent, trained investigators blinded to the clinical data. The QT was measured in all leads from the onset of the QRS to the end of the T-wave. In the presence of U wave, QT was measured to the nadir of the curve between the T and U-waves (14). QTd was defined as the difference between the maximum and minmum QT intervals and was obtained using Bazett's formula (6). Using 50 randomly chosen ECGS assessed the inter-rater and intra-rater variability. Both readers were blinded to all previous measurements.

### Clinical Outcome

The patients were followed up in person at 12 months. For patients with non-fatal events, we either called them back for a face-to-face follow-up or performed a home visit. The functional outcome status (modified Rankin Scale [mRS] scores ≥3) at 1 year after onset was the primary outcome of this study. Secondary efficacy outcomes included a new composite vascular event (ischemic stroke, hemorrhagic stroke, myocardial infarction, or vascular death) and stroke recurrene. Stroke recurrence was defined as a new stroke event (ischemic or hemorrhagic) accompanied by evidence of a stroke on magnetic resonance imaging or computed tomography of the brain.

### Statistical Analysis

Continuous variables with skewed distributions are presented using medians (interquartile ranges [IQR]) and those with normal distribution are presented using the mean (standard deviation [SD]). Categorical variables are described using percentages (%). Student's *t*-test and the Kruskal–Wallis rank test were used to compare parametric and non-parametric continuous variables, respectively. The chi-squared and Fisher's exact tests were used to compare qualitative data. Logistic regression analysis was performed to explore the independent predictors of QTd on functional outcome at 1 year. We assessed the associations between QTd and recurrent stroke and CVE using multivariable Cox regression models. The crude and multiple-adjusted odds ratios(OR) or adjusted hazard ratios (HRs) and their 95% confidence intervals (CIs) were calculated according to the quartile of QTd, using the lowest group as the reference. Variables with a *p* < 0.20 and the well-established predictors were selected as confounding variables into the multivariable analyses. The covariates included in the multivariable model were age, sex, habitual smoking, habitual drinking, medical history (hypertension, diabetes, dyslipidemia), and initial stroke severity (NIHSS), body mass index, ECG-LVH, Heart failure, Coronary heart disease, qualifying event, and secondary medication (anti-platelet, anti-lipid, and anti-hypertension), ischemic stroke subtype. SAS software, version 9.4 (SAS Institute, Inc, Cary, NC), was used for all statistical analyses, and two-sided *p* < 0.05 were considered statistically significant.

### Sensitivity Analyses

Additionally, we performed additional multivariable logistic regression analyses in patients without history of cardiovascular disease to eliminate the potential impact of cardiovascular disease on QTd.

## Results

### Characteristics of Patients

**Figure 1:**
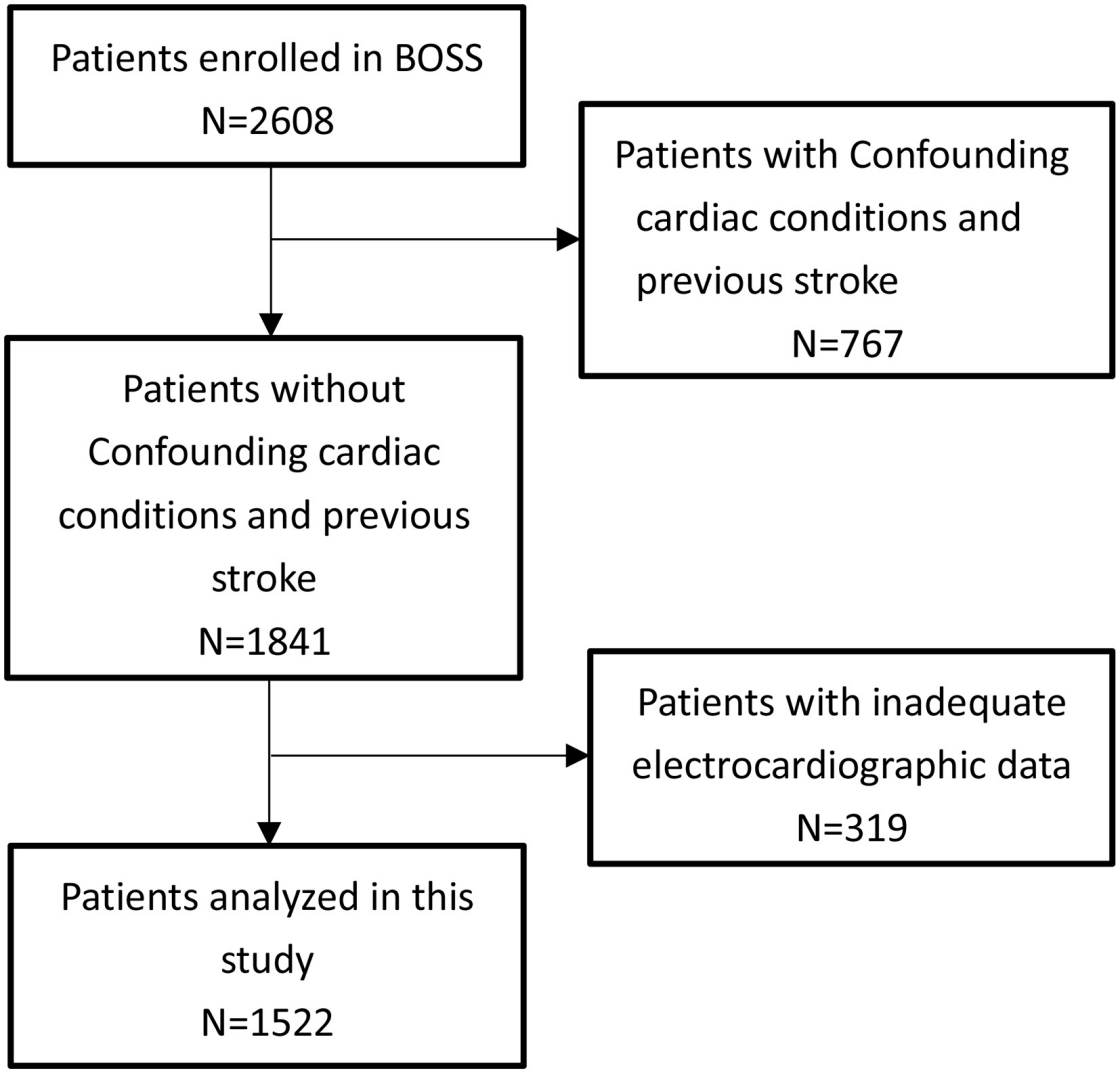
Flowchart of the study.

Of 1,841 IS patients, 319 patients with inadequate electrocardiographic were excluded. Therefore, 1,522 participants were included in the final analysis (Figure 1). The excluded population was more likely to have a history of hypertension, hyperlipidemia, and diabetes compared with the study population. Of the 1,522 included patients, 160 (10.51%) were TIA, the mean age was 61 ± 10 years, and 483 (31.73%) were female. The NIHSS score at presentation was 2 (1–4). The prevalence of major vascular risk factors was as follows: hypertension, 65.57%; diabetes, 20.30%; dyslipidemia, 8.21%; and habitual smoking, 44.09%. The mean QTd across all cases was 57 ms (40–83) (Table 1). Inter-rater reliability were ICC = 0.94 and intra-rater reliability were ICC = 0.95.

**Table 1:** Baseline characteristics of patients included vs. excluded.

**Characteristic**	**Total (*n* = 2,608)**	**Analyzed (*n* = 1,522)**	**Excluded (*n* =1,086)**	***P*-value**
Age, years, median (IQR)	63.00 (55.00, 71.00)	61.00 (54.00, 69.00)	64.00 (57.00, 73.00)	<0.0001
Male, *n* (%)	1,763 (67.60)	1,039 (68.27)	724 (66.67)	0.39
Smoking, *n* (%)	1,124 (43.1)	671 (44.09)	453 (41.71)	0.23
Drinking, *n* (%)	975 (37.38)	595 (39.09)	380 (34.99)	0.03
Diabetes, *n* (%)	568 (21.78)	309 (20.3)	259 (23.85)	0.03
Hypertension, *n* (%)	1,837 (70.44)	998 (65.57)	839 (77.26)	<0.0001
Hyperlipidemia, *n* (%)	265 (10.16)	125 (8.21)	140 (12.89)	<0.0001
ECG-LVH	100 (4.51)	68 (4.46)	32 (4.64)	0.84
Heart failure	20 (0.77)	6 (0.39)	14 (1.3)	0.009
Coronary heart disease	291 (11.16)	140 (9.17)	151 (13.97)	<0.0001
QTd, milliseconds median (IQR)	57.73 (40.33, 83.32)	57.70 (40.00, 83.26)	57.73 (40.99, 84.29)	0.58
Admission NIHSS, median (IQR)	2 (1, 4)	2 (1, 4)	2 (1, 5)	0.002
Qualifying event				0.52
IS	2,336 (89.81)	1,362 (89.49)	974 (90.27)	
TIA	265 (10.19)	160 (10.51)	105 (9.73)	
**Secondary medication**				
Anti-platelet	2,434 (96.28)	1,444 (97.70)	990 (94.29)	<0.0001
Anti-hypertension	1,731 (68.47)	981 (66.37)	750 (71.43)	0.007
Anti-lipid	2,196 (86.87)	1,293 (87.48)	903 (86.00)	0.28
Ischemic stroke subtype				<0.0001
Large-artery atherosclerosis	1,361 (52.19)	811 (53.11)	550 (50.88)	
Cardioembolism	83 (3.18)	11 (0.72)	72 (6.66)	
Small-artery occlusion	788 (30.21)	479 (31.37)	309 (28.58)	
Others	376 (14.42)	226 (14.80)	150 (13.88)	

*IQR, interquartile range; NIHSS, National Institutes of Health Stroke Scale*.

### QTd and Clinical Outcomes

During the 1 year of follow-up, 93 (6.1%) patients were lost. Functional dependency or death was documented in 214 (14.98%) cases at 1 year (Table 2). A total of 95 (6.24%) patients with CVE and 80 (5.26%) patients with recurrence stroke were identified at 1 year.

**Table 2:** Primary and secondary outcomes according to QTd quartiles.

**Variables**	**QTd quartiles (milliseconds)**				***p*-value**
	**Q1 (*n* = 375) 23.09 (19.14, 32.86)**	**Q2 (*n* = 386) 45.01 (42.58, 50.0)**	**Q3 (*n* = 379) 67.97 (62.92, 75.89)**	**Q4 (*n* = 382) 103.27 (89.44,122.16)**	
**OUTCOMES AT 1 YEAR**					
mRS score ≥3	44 (12.22)	52 (14.17)	48 (13.75)	70 (19.83)	0.026
Recurrent stroke	18 (4.8)	23 (5.9)	16 (4.2)	23 (6.0)	0.614
Composite vascular events	21 (5.6)	24 (6.2)	19 (5.0)	31 (8.1)	0.317

*mRS, modified Rankin Scale; composite vascular events include recurrent stroke, myocardial infarction, and vascular death after the index stroke*.

**Figure 2:**
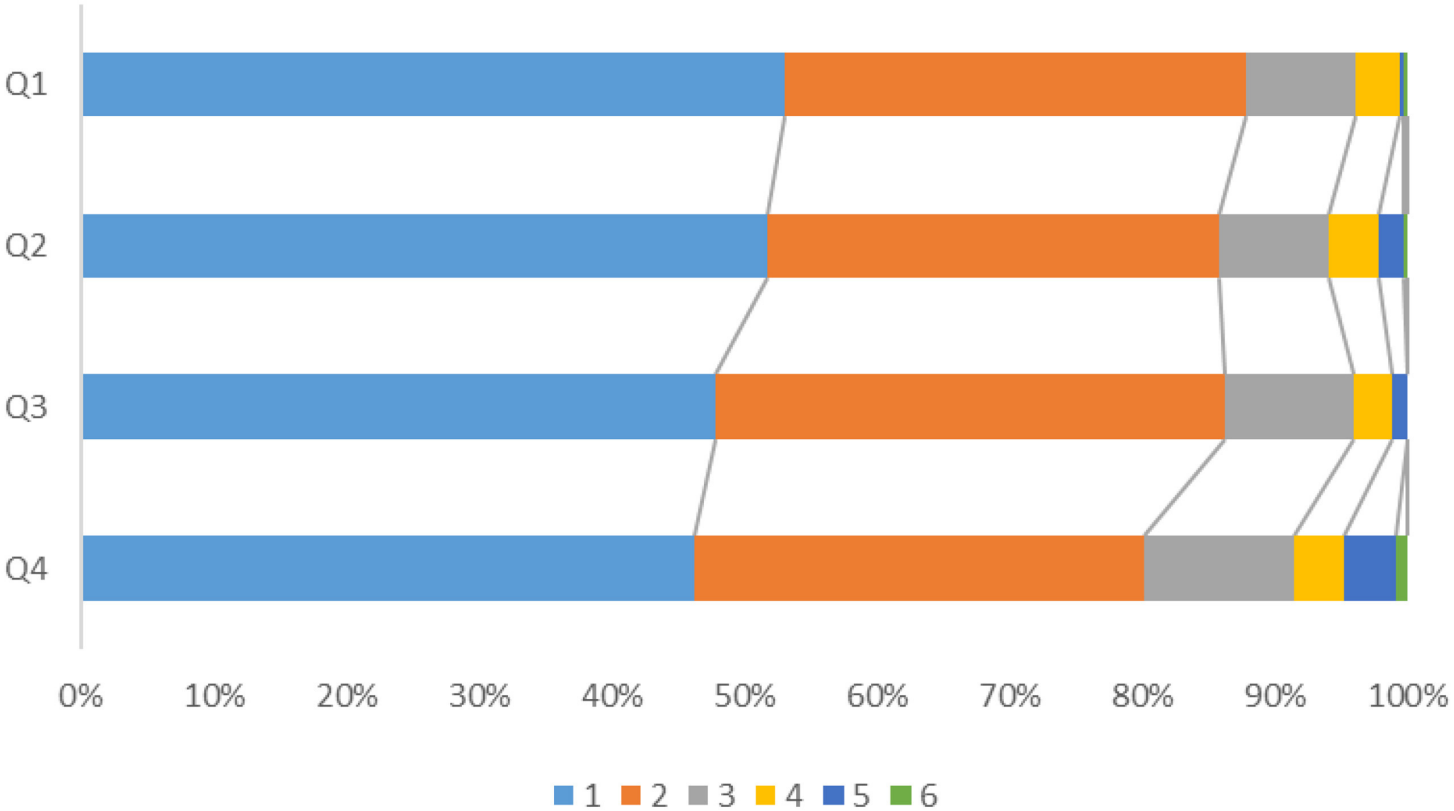
Modified Rankin Scale (mRS) at 1 year according to QTd quartile.

**Figure 3:**
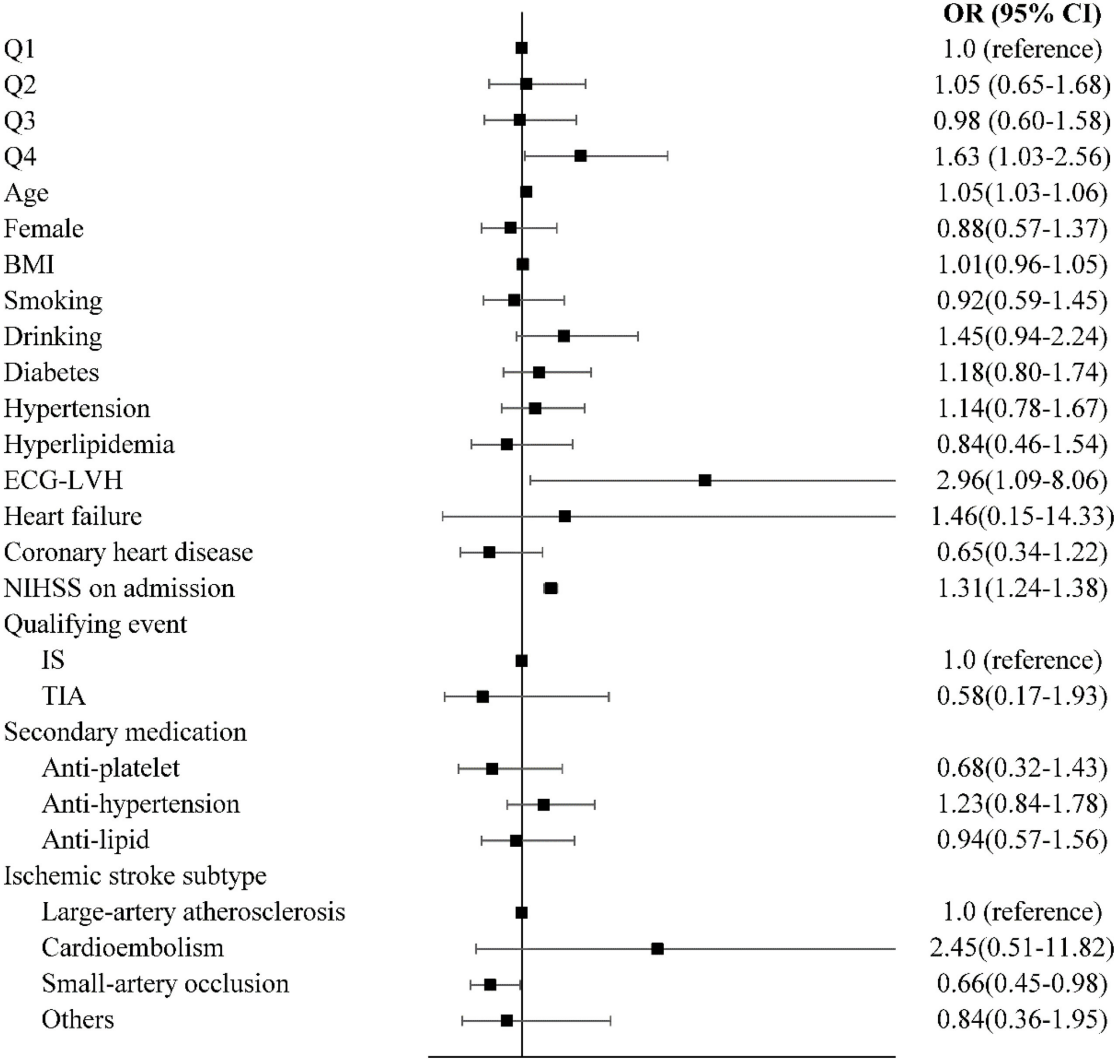
Multivariable logistic regression analyses between QTd and functional recovery. OR, odds ratio; CI, confidence interval; mRS, modified Rankin Scale.

After adjusting for confounders, the prevalence of death and major disability (mRS ≥ 3) showed significant differences according to the quartile of QTd (Figure 2), with the risk of death and major disability (mRS ≥ 3) at 1 year being significantly higher for patients in Q4 than for those in Q1 (adjusted OR = 1.626, 95% CI:1.033–2.560) (Figure 3). However, there were no significant correlation between QTd and the event outcomes at 1 year.

### Sensitivity Analyses

A sensitivity analysis of patients without cardiovascular disease was conducted, and the results showed that patients in Q4 still had higher risks for functional dependence or death compared with the Q1 group (Table 3).

**Table 3:** Sensitivity analyses of association of QTd with functional outcome (mRS ≥ 3) at 1 year.

	**Odds Ratios (95% Confidence Interval [CI])**		
	**Unadjusted**	Model 1^*^ (95% CI)	**Model 2^†^ (95% CI)**
Q1	1.0 (reference)	1.0 (reference)	1.0 (reference)
Q2	1.23 (0.78–1.93)	1.28 (0.81–2.02)	1.13 (0.68–1.86)
Q3	1.17 (0.73–1.85)	1.15 (0.72–1.84)	1.02 (0.61–1.70)
Q4	1.81 (1.17–2.79)	1.81 (1.16–2.80)	1.72 (1.06–2.79)

* *Model 1: adjusted for age and sex*.

† *Model 2: adjusted for age, sex, hypertension, diabetes, hyperlipidemia, smoking, drinking, and national institutes of health stroke scale on admission. Body mass index, qualifying event, and secondary medication (anti-platelet, anti-lipid, and anti-hypertension), ischemic stroke subtype*.

## Discussion

In this prospective cohort study, we found that an increase in QTd is associated, in a magnitude-dependent fashion, with an increase in poor functional outcomes at 12 months in IS or TIA patients which was independent of age, NIHSS, other co-morbidities, and other cardiovascular risk factors.

Cardiovascular complications and several ECG abnormalities unrelated to ischemic heart disease are extremely common following stroke and represent a major form of morbidity (15). Disturbances in the autonomic nervous system are thought to be responsible for these cardiac electrophysiological changes (16). QTd has been suggested to be useful as an indirect method of measuring cardiac repolarization abnormalities in the recent 20 years. However, there have been conflicting results on the prognostic value of QTd in patients with ischemic stroke.

Our results were consistent with those of most previous studies. A prospective study of 93 hemorrhagic stroke patients found that a higher QT_max_ had a positive relationship to bed confinement and Barthel index of 28 days (17). The same result was found in a observational case control study which showed prolonged corrected QT interval (QTc) in acute IS patients 48 h after the index stroke correlated with admission NIHSS and discharge mRS scores (12). Meanwhile, Stead et al. found no relationship between prolonged QTc and discharge mRS (18). Furthermore, a prospective cohort study provided no evidence that QTd is related to high mortality and poor functional outcomes on hospital discharge among acute IS patients (19). The US Third National Health and Nutrition Examination Survey suggested that a prolonged corrected QT interval can predict all-cause mortality in the general population (20). The REasons for Geographic and Racial Differences in Stroke (REGARDS) Study showed that patients with prolonged QT have a higher risk of IS (21). In addition to supporting the findings of these previous studies, our results also suggest that QTd is a predictor of adverse functional outcome even up to 1 year later. Despite the differing results in the above studies, we believe that the present study strengthens the evidence for the relationship between QT interval and clinical outcomes in an IS/TIA population.

As we all known that the increase in QTd during the acute phase probably due to excessive circulating catecholamines secreted by the hypothalamic-pituitary-adrenal (HPA) axis, as well as massive catecholamine release from myocardial nerve endings (16). We speculated that QTd can aid in identifying subclinical cardiac disease, as well as reversible acute stress cardiomyopathy induced by cerebral injury, likely mediated by autonomic nervous dysfunction and increased circulating catecholamines. Some evidence from diabetic patients shows that an increase in the number of cardiac abnormalities increases the prolongation of QTd (22). One study found that acute cerebral lesions cause abnormally high levels of plasma catecholamines which can result in cardiac repolarization abnormalities and stress cardiomyopathy, but not myocardial infarction (23). Another study showed that the sympathetic nerve can directly release catecholamines at myocardial nerve endings and thereby induce cardiomyocyte toxicity (24). Cardiac dysfunction reflected in QTd may compromise perfusion to the brain sufficiently to damage the ischemic zone of the brain and poor prognosis may occur (25, 26).

The congenital long QT syndrome is an inheritable ion channelopathy that has traditionally been considered to be a purely electrical disease. However, using various imaging techniques, mechanical alterations have recently been reported: differences in mechanical and electrical timing; a reduction in both systolic and diastolic function (27); as well as a shorter left ventricular filling time and smaller stroke volume (28). Thus, we speculated that an abnormal QT interval is not just an electrical phenomenon, but also an electromechanical one that may be a potential pathomechanism for adverse events and may contribute to risk stratification of IS patients.

In this large, prospective cohort study, QTd (an established ECG marker of left ventricular abnormality) was significantly associated with poor functional outcomes regardless of confounding factors. Few studies that examine the prognostic value of QTd in a non-Western acute stroke population have been performed. However, this study also has some limitations. First, we were unable to collect pre-stroke ECGs, which would have allowed us to exclude the possibility that the abnormal QTd values were caused by preexisting heart disease, or Serial QTd measurements, which would have evaluated exactly the correlation between stroke outcomes and QTd. To mitigate this, we performed a sensitivity analyses. It showed that subgroups without history of cardiovascular disease also exhibited a significant correlation between QTd and study outcomes. Therefore, acute cerebrovascular disease itself may be the main cause of the increased QTd of our patients. Second, There may be a selection bias because the cohort we studied had no history of stroke indicating that the results may not generalize to all stroke patients. Third, We are short of information about the drugs and rehabilitation interventions out of the hospital which may affects the robustness of the main analysis. Fourth, We had no control for confounding factors of medications or serum electrolytes which may lead to alteration of the QT interval. Fifth, We studied a cohort of patients with low NIHSS scores, whether our findings can be extrapolated to more severe patients safely is not known. Last, As in other epidemiologic studies, residual confounding remains a possibility, although we adjusted for several factors which may affect the prognosis of IS.

## Conclusions

An increase in QTd was associated with poorer functional outcomes up to 1 year after the index event. These results showed that electrocardiographic assessment for cases with stroke may provide significant prognostic information for patients and pathogenetic perception in restoration of function. QTd is a useful surrogate marker of adverse functional prognosis that provides effective risk stratification of cerebrovascular disease.

## Data Availability Statement

The raw data supporting the conclusions of this article will be made available by the authors, without undue reservation.

## Ethics Statement

The study was approved by the Institutional Review Board at Beijing Tiantan Hospital, as well as ethical committees at the participating hospitals, in compliance with the Declaration of Helsinki. All patients or their legal authorized representatives provided written informed consent before participation.

## Author Contributions

HT designed and wrote the manuscript. AW and YZ performed the data analysis. YoW revised the manuscript. All authors contributed toward data analysis, drafting, and critically revising the article.

## Conflict of Interest

The authors declare that the research was conducted in the absence of any commercial or financial relationships that could be construed as a potential conflict of interest.

## References

[1] Caplan LR Fisher M. Personalised care of patients with stroke in China: a challenge and an opportunity. Stroke Vasc Neurol. (2016) 1:3–5. 10.1136/svn-2015-000005 28959459 PMC5435203

[2] Tu WJ Dong X Zhao SJ Yang DG Chen H. Prognostic value of plasma neuroendocrine biomarkers in patients with acute ischemic stroke. J Neuroendocrinol. (2013) 25:771–8. 10.4414/smw.2010.13101 23701638

[3] Zheng H Cao N Yin Y Feng W. Stroke recovery and rehabilitation in 2016: a year in review of basic science and clinical science. Stroke Vasc Neurol. (2017) 2:222–9. 10.1136/svn-2017-000069 29507783 PMC5829939

[4] Ntaios G Papavasileiou V Michel P Tatlisumak T Strbian D. Predicting functional outcome and symptomatic intracranial hemorrhage in patients with acute ischemic stroke: a glimpse into the crystal ball? Stroke. (2015) 46:899–908. 10.1161/strokeaha.114.003665 25657189

[5] Wong KY Mac Walter RS Douglas D Fraser HW Ogston SA Struthers AD. Long QTc predicts future cardiac death in stroke survivors. Heart. (2003) 89:377–81. 10.1136/heart.89.4.377 12639861 PMC1769252

[6] Lederman YS Balucani C Lazar J Steinberg L Gugger J Levine SR. Relationship between QT interval dispersion in acute stroke and stroke prognosis: a systematic review. J Stroke Cerebrovasc Dis. (2014) 23:2467–78. 10.1016/j.jstrokecerebrovasdis.2014.06.004 25282188 PMC4256166

[7] Munro SF Cooke D Kiln-Barfoot V Quinn T. The use and impact of 12-lead electrocardiograms in acute stroke patients: a systematic review. Eur Heart J Acute Cardiovasc Care. (2018) 7:257–63. 10.1177/2048872615620893 26637212

[8] Fukui S Katoh H Tsuzuki N Ishihara S Otani N Ooigawa H. Multivariate analysis of risk factors for QT prolongation following subarachnoid hemorrhage. Crit Care. (2003) 7:R7–12. 10.1186/cc2160 12793884 PMC270671

[9] Lazar J Manzella S Moonjelly J Wirkowski E Cohen TJ. The prognostic value of QT dispersion in patients presenting with acute neurological events. J Invasive Cardiol. (2003) 15:31–35. 12499526

[10] Huang CH Chen WJ Chang WT Yip PK Lee YT. QTc dispersion as a prognostic factor in intracerebral hemorrhage. Am J Emerg Med. (2004) 22:141–4. 10.1016/j.ajem.2004.02.029 15138946

[11] Lazar J Busch D Wirkowski E Clark LT Salciccioli L. Changes in QT dispersion after thrombolysis for stroke. Int J Cardiol. (2008) 125:258–62. 10.1016/j.ijcard.2007.03.114 17509702

[12] Hromádka M Seidlerová J Rohan V Baxa J Šedivý J Rajdl D. Prolonged corrected QT interval as a predictor of clinical outcome in acute ischemic stroke. J Stroke Cerebrovasc Dis. (2016) 25:2911–7. 10.1016/j.jstrokecerebrovasdis.2016.08.005 27618199

[13] Xu J Liu Y Tao Y Xie X Gu H Pan Y The design, rationale, and baseline characteristics of a nationwide cohort registry in China: blood pressure and clinical outcome in TIA or ischemic stroke. Patient Prefer Adherence. (2016) 10:2419–27. 10.2147/ppa.s119825 27942205 PMC5138037

[14] Lepeschkin E Surawicz B. The measurement of the Q-T interval of the electrocardiogram. Circulation. (1952) 6:378–88. 10.1161/01.cir.6.3.378 14954534

[15] Jiang B Han X Wang L Dong Q. Prognosis of early-stage continuous electrocardiogram abnormalities on patients with acute ischemic stroke. J Stroke Cerebrovasc Dis. (2015) 24:1761–7. 10.1016/j.jstrokecerebrovasdis.2015.03.043 25939863

[16] Chen Z Venkat P Seyfried D Chopp M Yan T Chen J. Brain-heart interaction: cardiac complications after stroke. Circ Res. (2017) 121:451–68. 10.1161/circresaha.117.311170 28775014 PMC5553569

[17] Chao CC Wang TL Chong CF Lin YM Chen CC Tang GJ. Prognostic value of QT parameters in patients with acute hemorrhagic stroke: a prospective evaluation with respect to mortality and post-hospitalization bed confinement. J Chin Med Assoc. (2009) 72:124–32. 10.1016/s1726-4901(09)70037-1 19299219

[18] Stead LG Gilmore RM Bellolio MF Vaidyanathan L Weaver AL Decker WW. Prolonged QTc as a predictor of mortality in acute ischemic stroke. J Stroke Cerebrovasc Dis. (2009) 18:469–74. 10.1016/j.jstrokecerebrovasdis.2009.02.006 19900651

[19] Lederman YS Balucani C Steinberg LR Philip C Lazar JM Weedon J. Does the magnitude of the electrocardiogram qt interval dispersion predict stroke outcome? J Stroke Cerebrovasc Dis. (2019) 28:44–8. 10.1016/j.jstrokecerebrovasdis.2018.09.006 30291031

[20] Soliman EZ Shah AJ Boerkircher A Li Y Rautaharju PM. Inter-relationship between electrocardiographic left ventricular hypertrophy and QT prolongation as predictors of increased risk of mortality in the general population. Circ Arrhythm Electrophysiol. (2014) 7:400–6. 10.1161/circep.113.001396 24762807 PMC4314284

[21] O'Neal WT Howard VJ Kleindorfer D Kissela B Judd SE McClure LA. Interrelationship between electrocardiographic left ventricular hypertrophy, QT prolongation, and ischaemic stroke: the reasons for geographic and racial differences in stroke study. Europace. (2016) 18:767–72. 10.1093/europace/euv232 26487665 PMC4880112

[22] Rana BS Band MM Ogston S Morris AD Pringle SD Struthers AD. Relation of QT interval dispersion to the number of different cardiac abnormalities in diabetes mellitus. Am J Cardiol. (2002) 90:483–7. 10.1016/s0002-9149(02)02518-3 12208406

[23] Abraham J Mudd JO Kapur NK Klein K Champion HC Wittstein IS. Stress cardiomyopathy after intravenous administration of catecholamines and beta-receptor agonists. J Am Coll Cardiol. (2009) 53:1320–5. 10.1016/j.jacc.2009.02.020 19358948

[24] Mertes PM Carteaux JP Jaboin Y Pinelli G el Abassi K Dopff C. Estimation of myocardial interstitial norepinephrine release after brain death using cardiac microdialysis. Transplantation. (1994) 57:371–7. 10.1097/00007890-199402150-00010 8108872

[25] Milionis H Faouzi M Cordier M D'Ambrogio-Remillard S Eskandari A Michel P. Characteristics and early and long-term outcome in patients with acute ischemic stroke and low ejection fraction. Int J Cardiol. (2013) 168:1082–7. 10.1016/j.ijcard.2012.11.036 23176765

[26] Lee EJ Noh SM Kang DW Kim JS Kwon SU. Impact of provoking risk factors on the prognosis of cerebral venous thrombosis in Korean patients. J Stroke. (2016) 18:187–94. 10.5853/jos.2015.01669 27165266 PMC4901946

[27] Leren IS Hasselberg NE Saberniak J Haland TF Kongsgard E Smiseth OA. Cardiac mechanical alterations and genotype specific differences in subjects with long QT syndrome. JACC Cardiovasc Imaging. (2015) 8:501–10. 10.1016/j.jcmg.2014.12.023 25890583

[28] Charisopoulou D Koulaouzidis G Rydberg A Henein MY. Abnormal ventricular repolarization in long QT syndrome carriers is related to short left ventricular filling time and attenuated stroke volume response during exercise. Echocardiography. (2018) 35:1116–23. 10.1111/echo.13891 29648704

